# Predator discrimination of prey promotes the predator-mediated coexistence of prey species

**DOI:** 10.1098/rsos.220859

**Published:** 2022-12-07

**Authors:** Gen Iwashita, Akira Yamawo, Michio Kondoh

**Affiliations:** ^1^ Graduate School of Life Sciences, Tohoku University Japan, Sendai 980-8577, Japan; ^2^ Department of Biology, Faculty of Agriculture and Life Science, Hirosaki University, Hirosaki Aomori 036-8561, Japan

**Keywords:** apparent competition, multi-species coexistence, adaptive foraging, foraging theory, prey switching, cognitive science

## Abstract

The predator discrimination of prey can affect predation intensity and the prey density dependence of predators, which has the potential to alter the coexistence of prey species. We used a predator–prey population dynamics model accounting for the predator's adaptive diet choice and predator discrimination of prey to investigate how the latter influences prey coexistence. The model revealed that (i) prey species that are perceived as belonging to the same species by a predator are attacked in the same manner, and it is more difficult for them to coexist than those that are recognized as different prey species, and (ii) prey species that are not discriminated by a predator—and therefore cannot coexist—may coexist in the presence of an alternative predator that does discriminate between them. These results suggest that prey diversity, which favours the predator discrimination of prey, and the different capabilities of predators to identify prey species both enhance prey coexistence.

## 1. Introduction

An understanding of the mechanisms of multi-species coexistence is a central question in community ecology [1]; however, one of its most important aspects is interspecific interaction. Predator species have a crucial influence on the potential multi-species coexistence of prey species [2–4]. The impact of predator species on prey communities is dependent on the magnitude of predation pressure and its response to changing prey density [5–7]. Indeed, the two major mechanisms through which a predator species affects prey coexistence are associated with the responses of the predator's density (numerical response) and the predator's behaviour (functional response [8,9]) to changes in prey density. Apparent competition, which is the negative interaction that emerges in prey species with a shared predator [3,10], is explained by a numerical response in which an increased density in one prey species leads to an increase in the density of the shared predator. The predator's preference for more abundant prey in higher population densities switching predation induces prey coexistence [5,11,12]. An explanation for this is the functional response of predators that select the most abundant prey, which results in negative frequency-dependent selection. To understand the role of predators in mediating prey coexistence, it is essential to consider both the numerical and functional responses.

When combined with the adaptive diet-selection behaviour, which optimizes the combination of prey species such that the predator maximizes the gain from predation, the manner in which the predator species distinguish between their prey species is likely to affect the shape of the functional response and, consequently, prey population dynamics and coexistence. The theories of optimal foraging [13] and adaptive foraging [14] assume that predators choose prey items in an optimal way to maximize the amount of gain per unit of time. The theory indicates that predators prefer prey items with a higher gain. However, it should be noted that the identity of a ‘prey item’ may not correspond to species identity. As reported by Holling [15], the predators' units of predator discrimination of prey do not always correspond to species [16–19]. In theoretical studies of Batesian mimicry [20], in which a palatable prey species resembles an unpalatable species, it is predicted that prey species that are indistinguishable to a predator species will have difficulty coexisting [21,22].

However, a theoretical investigation of the effect of predator discrimination on the coexistence of prey species is insufficient. In previous theoretical studies, mathematical models of mimicry considered the potential inability of predator species to identify their prey species by assuming perfect [21,23,24] or imperfect [22,25,26] mimicry. Given that prey species have multiple traits [27] and that predator species recognize prey items by various cues [16–19], it is possible that in an actual community, there may be diversity in how predators distinguish prey items. However, most mathematical models have not assumed interspecies diversity in the predator discrimination of prey. A more theoretical exploration of how the predator discrimination of prey affects the coexistence of prey species would require a mathematical model in which multiple predator species can have multiple methods for predator discrimination of prey.

In this study, a simple mathematical model of a multiple predator–prey community that explicitly incorporates predator discrimination of prey is presented and then used to investigate how the predator discrimination of prey affects the coexistence of prey species. As predator discrimination of prey does not necessarily indicate species discrimination, predators with adaptive foraging behaviour would exhibit the same behaviour toward prey species that they perceive as belonging to the same type of prey item, but which are actually different prey species. In this mathematical model, the effects of predator discrimination of prey type and its variation on prey coexistence were analysed. The results showed that both the manner in which predators distinguish prey species and the variability of predator discrimination of prey can affect the coexistence of prey species. Specifically, the coexistence of different prey species that are recognized as the same prey item is more difficult than the coexistence of prey species that are perceived as different items. Further, prey species that do not coexist in a situation where there is a predator with one method of discrimination will coexist when there is another predator that discriminates prey in a different manner.

## 2. Methods

The model considers a community of *N_C_* predator species, with a density *C_i_* (*i* = 1, … , *N_C_*), and *N_R_* prey species, with a density *R_j_* (*j* = 1, … , *N_R_*). A predator species perceives the prey community comprising several ‘prey groups’, among which the predator species can discriminate. In other words, the prey group of a given predator species is a set of prey species that the predator species cannot discriminate between. The species composition of prey groups may differ for each predator species. We do not assume that different parts of the same prey species belong to different prey groups for a given predator species (e.g. the situation that predator species distinguish individuals at larval stage from those at adult stage). Based on the assumption that prey species are not only limited by predator species, prey–predator population dynamics can be described by the following differential equations:

2.1 $$\frac{dC_{i}}{dt}=C_{i}\left(\overset{N_{i}}{\underset{k=1}{\sum }}a_{ik}\left(\underset{\;j\in K_{ik}}{\sum }\lambda_{ij}b_{j}R_{j}\right)-m_{i}\right)$$

and

2.2 $$\frac{dR_{j}}{dt}=R_{j}\left(r_{j}-s_{j}R_{j}-\overset{N_{c}}{\underset{j}{\sum }}a_{ij}\lambda_{ij}C_{i}\right),$$

where *N_i_* is the number of Predator *i*'s prey groups, *K_ik_* is the *k*th prey group for Predator *i*, *m_i_* is the mortality rate of Predator *i*, *r_j_* is the intrinsic growth rate of Prey *j*, *s_j_* is the intraspecific competition coefficient of Prey *i*, *b_j_* is the assimilation efficiency of Prey *j*, *λ_ij_* is the foraging efficiency of Predator *i* on Prey *j*, and *a_ik_* is the predation effort of Predator *i* on prey group *K_ik_*, which varies adaptively over time according to the following equations [5]:

2.3 $$P_{ik}=\underset{\;j\in K_{ik}}{\sum }\lambda_{ij}b_{j}R_{j},$$

2.4 $$\frac{da_{ik}}{dt}=Ga_{ik}\left(P_{ik}-\overset{N_{i}}{\underset{l=1}{\sum }}a_{il}P_{il}\right)$$

2.5 $$\mathrm{and}\;\;\;\overset{N_{i}}{\underset{k=1}{\sum }}a_{ik}=1,$$

where *P_ik_* is the food gain per unit effort from the *k*th prey group for Predator *i*, and *G* is the scaling parameter representing the relative speed of adaptive dynamics to population dynamics. The adaptive dynamics are represented by the replicator equation (2.4), which indicates that if the food gain per unit effort for a prey group is above average, the predator species effort for that group will be increased, whereas if it is lower, the foraging effort will be reduced. Prey species (*j*, *j′*) that are perceived by Predator *i* to belong to the same group are assumed to be subject to the same predation pressure (*a_ij_* = *a_ij′_*).

## 3. Results

We first considered the case in which the foraging behaviour of predators is faster than the population dynamics (*G* ≫ 1) [28]. This assumption means that predator species allocate optimal foraging efforts at the current prey species population.

We can prove the following proposition in the case in which the adaptive dynamics are faster than the other dynamics (electronic supplementary material, appendix S1).

### 3.1. Proposition 6

Assume that the dynamics of the predator's foraging effort are faster than the population dynamics (*G* ≫ 1), i.e. predator species allocate optimal foraging efforts at the current prey species population. In such a case, the equilibrium point of a community with *N_c_* predator species and *N_R_* prey species is asymptotically stable if the gain that each predator species receives from the used prey group is greater than the gain that the predator could receive from any unused prey groups.

First, a one-predator (*C*_1_)–two-prey (*R*_1_, *R*_2_) system was considered. It was assumed that the ratio *r*_1_/*λ*_11_ of the foraging efficiency of Predator 1 on Prey 1 (*λ*_11_) to the intrinsic growth rate of Prey 1 (*r*_1_) is greater than that of Prey 2 ($r_{1}/\lambda_{11}\geq r_{2}/\lambda_{12}$), without a loss of generality.

If the predator species distinguishes between the two prey species, the population dynamics models of the three species can be presented as follows:

3.1 $$\frac{dC_{1}}{dt}=C_{1}(a_{1}\lambda_{11}b_{1}R_{1}+a_{2}\lambda_{12}b_{2}R_{2}-m_{1}),$$

3.2 $$\frac{dR_{1}}{dt}=R_{1}(r_{1}-s_{1}R_{1}-a_{1}\lambda_{11}C_{1}),$$

3.3 $$\frac{dR_{2}}{dt}=R_{2}(r_{2}-s_{2}R_{2}-a_{2}\lambda_{12}C_{1})$$

3.4 $$\mathrm{and}\;\;a_{1}+a_{2}=1,$$

where *a_i_* is Predator 1's foraging effort for the prey group *i* (Prey *i*), *m*_1_ is the mortality rate of Predator 1, *r_i_* is the intrinsic growth rate of Prey *i*, *s* is the intraspecific competition coefficient of prey species, *b_i_* is the assimilation efficiency of Prey *i*, and *λ_ij_* is the foraging efficiency of Predator *i* on Prey *j*.

This model is of the same form as the previous study [29]. In this case, two prey species will always stably coexist at the equilibrium point (see electronic supplementary material, appendix S2).

If the predator species recognizes the two prey species as belonging to the same prey group, the following model for the population dynamics of the three species is applicable:

3.5 $$\frac{dC_{1}}{dt}=C_{1}(b_{1}\lambda_{11}R_{1}+b_{2}\lambda_{12}R_{2}-m_{1})$$

and

3.6 $$\frac{dR_{i}}{dt}=R_{i}(r_{i}-s_{i}R_{i}-\lambda_{1i}C_{1})\;(i=1,2),$$

where *m*_1_ is the mortality rate of Predator 1, *r_i_* is the intrinsic growth rate of Prey *i*, *s* is the intraspecific competition coefficient of prey species, *b_i_* is the assimilation efficiency of Prey *i*, and *λ*_1*j*_ is the foraging efficiency of Predator 1 on Prey *i*.

In this case, the three species will stably coexist in equilibrium if, and only if, the following condition is satisfied (see electronic supplementary material, appendix S3):

3.7 $$\frac{r_{1}}{\lambda_{11}}-\frac{r_{2}}{\lambda_{12}}<\frac{m_{1}s_{1}}{b_{1}\lambda_{11}^{2}}.$$

Inequality (3.7) suggests that two prey species with largely different growth rates cannot coexist because of apparent competition [3,8]. More specifically, a prey species with a higher growth rate excludes the other prey species through the indirect negative effect mediated by the predator species.

Next, a community with one predator (*C*_1_) and three prey species (*R*_1_, *R*_2_, *R*_3_) was considered. The predator species recognizes Prey 1 and Prey 2 as belonging to the same group. Working on the assumption that the ratio $r_{1}/\lambda_{11}$ of the foraging efficiency of Predator 1 on Prey 1 (*λ*_11_) to the intrinsic growth rate of Prey 1 (*r*_1_) is greater than that of Prey 2 ($r_{1}/\lambda_{11}\geq r_{2}/\lambda_{12}$), the population dynamics model can be written as follows:

3.8 $$\frac{dC_{1}}{dt}=C_{1}(a_{1}P_{1}+a_{2}P_{2}-m_{1}),$$

3.9 $$\frac{dR_{i}}{dt}=R_{i}(r_{i}-s_{i}R_{i}-a_{i}\lambda_{1j}C_{1}),$$

3.10 $$P_{1}=b_{1}\lambda_{11}R_{1}+b_{2}\lambda_{12}R_{2},$$

3.11 $$P_{2}=b_{3}\lambda_{13}R_{3}$$

3.12 $$\mathrm{and}\;\;\;a_{1}+a_{2}=1,$$

where *a_k_* is Predator 1's foraging effort for prey group *k*, *P_k_* is the food gain per unit effort from the *k*th prey group for Predator 1, *m*_1_ is the mortality rate of Predator 1, *r_i_* is the intrinsic growth rate of Prey *i* (*r*_1_ ≥ *r*_2_), *s_i_* is the intraspecific competition coefficient of Prey *i*, *b_i_* is the assimilation efficiency of Prey *j*, and *λ_ij_* is the foraging efficiency of Predator 1 on Prey *j*.

In this case, the condition for the stable coexistence of the three prey species at the equilibrium point is again given by inequality (3.7); if the condition does not hold and Predator 1 eats prey group 1 at the equilibrium point, only Prey 1 and Prey 3 coexist, while Prey 2 is excluded (see electronic supplementary material, appendix S4).

This indicates that (i) prey species that are discriminated between by Predator 1 can always coexist; and (ii) if the strength of apparent competition between two prey species that Predator 1 recognizes as identical is weak, then three prey species can coexist (inequality (3.7)).

**Figure 1.**
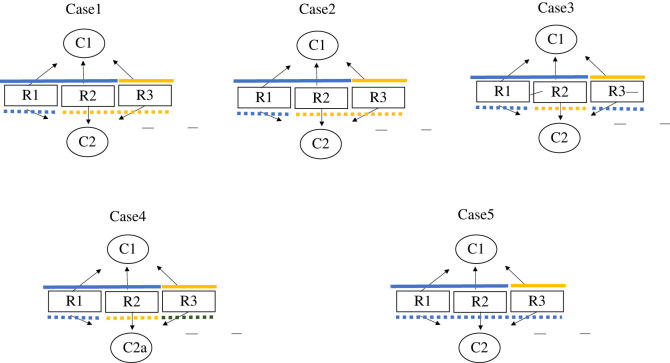
Five cases of predator discrimination of prey by predators. Two predator species (C1 and C2) potentially use three prey species (R1, R2 and R3). While C1 discriminates a prey group consisting of R1 and R2 from the other group that includes R3, C2 discriminates prey in five different ways. The blue, yellow and green lines indicate the grouping adopted by C1 (solid lines) and C2 (dotted lines).

In the case that prey species cannot coexist when Predator 1 is present, what happens to the coexistence of prey species when another predator (Predator 2: *C*_2_) exists was examined. The following five cases were explored (figure 1):

Case 1: Predator 2 recognizes Prey 1 and Prey 2 as identical (belonging to the same prey group);
Case 2: Predator 2 recognizes Prey 2 and Prey 3 as identical;
Case 3: Predator 2 recognizes Prey 1 and Prey 3 as identical;
Case 4: Predator 2 distinguishes all the prey species;
Case 5: Predator 2 cannot distinguish any prey species.

In all cases, the population dynamics of the predator–prey relationship and the adaptive dynamics of predation effort can be written as follows:

3.13 $$\frac{dC_{1}}{dt}=C_{1}(a_{11}P_{11}+a_{12}P_{12}-m_{1}),$$

3.14 $$\frac{dC_{2}}{dt}=C_{2}\left(\overset{N_{k}}{\underset{k}{\sum }}a_{2k}P_{2k}-m_{2}\right),$$

3.15 $$P_{11}=b_{1}\lambda_{11}R_{1}+b_{2}\lambda_{12}R_{2},\;P_{12}=b_{3}\lambda_{13}R_{3},$$

3.16 $$P_{2k}=\overset{N_{2k}}{\underset{l}{\sum }}b_{l}\lambda_{2l}R_{l},$$

3.17 $$\frac{dR_{j}}{dt}=R_{j}(r_{j}-s_{j}R_{j}-a_{1l}\lambda_{1l}C_{1}-a_{2k}\lambda_{2l}C_{2})$$

3.18 $$\mathrm{and}\;a_{11}+a_{12}=1,\overset{N_{k}}{\underset{k}{\sum }}a_{2k}=1,$$

where *m_i_* is the mortality rate of Predator *i*, *r_j_* is the intrinsic growth rate of Prey *j*, *s_j_* is the intraspecific competition coefficient of Prey *j*, *b_j_* is the assimilation efficiency of Prey *j*, *λ_ij_* is the foraging efficiency of Predator *i* on Prey *j, a_ik_* is the predation effort of Predator *i* on prey group, *N*_2*k*_ is the *k*th prey group for Predator *2*, *P_ik_* is the food gain per unit effort from the *k*th prey group for Predator *i*, and *N_k_* represents the number of Predator 2's prey groups.

To examine the effect of predator discrimination of prey on the coexistence of prey species, we assume that the only difference between Predator 2 and Predator 1 is how they discriminate between prey species. Moreover, we assume that the foraging efficiency (*λ*) is the same for all predator species. The following relationship holds:

3.19 $$\lambda_{1l}=\lambda_{2l}.$$

Analyses showed that the three prey species stably coexist at the equilibrium point in the presence of two predator species in all cases except for Case 1 and Case 5 (see electronic supplementary material, appendix S5). Even if the apparent competition between prey species (Prey 1 and Prey 2), which Predator 1 recognizes as the same prey item, is too strong for coexistence, coexistence may be possible when another predator (Predator 2) discriminates these prey species, as in Case 2, Case 3 and Case 4.

**Figure 2.**
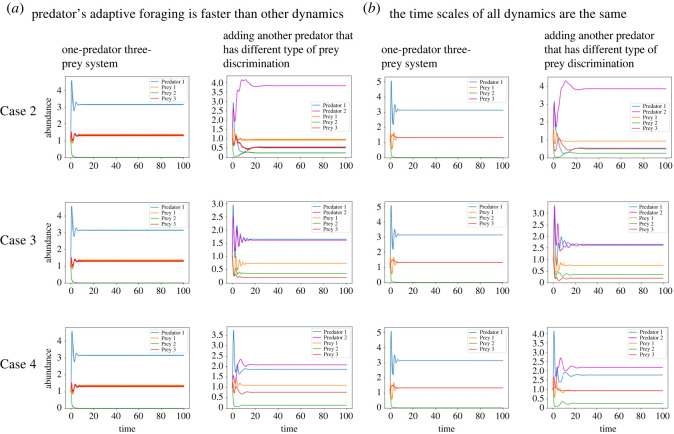
Population dynamics of the two-predator–three-prey model. When the foraging behaviour of the predator is on the same time scale as the other dynamics (*b*), predator species that differ in their predator discrimination of prey facilitate the coexistence of the three prey species, as do time scales in which foraging behaviour is faster than population dynamics (*a*). The initial conditions of (C_1_, C_2_, R_1_, R_2_, R_3_, a_11_, a_12_, a_21_, a_22_) are (1, 1, 1, 1, 1, 0.5, 0.5, 0.5, 0.5) for Cases 2 and 3, respectively, and the initial condition of (C_1_, C_2_, R_1_, R2, R_3_, a_11_, a_12_, a_21_, a_22_, a_23_) is (1, 1, 1, 1, 1, 0.5, 0.5, 0.4, 0.3, 0.3, 1, 1, 1, 1, 1, 1) for Case 4. The other parameters are (*r*_1_, *r*_2_, *r*_3_, b_1_, b_2_, b_3_, m_1_, m_2_, *λ*_11_, *λ*_12_, *λ*_13_, *λ*_21_, *λ*22, *λ*_23_) = (4, 2, 1.8, 1.5, 2.5, 1.5, 2, 1.4, 1, 1, 1, 1, 1, 1), for more details see electronic supplementary material, appendix S6.

We then considered the case in which foraging behaviour occurs on the same time scale as other dynamics (*G* = 1). In this case, the foraging behaviour of predator species is given by equation (2.4). We simulated the one-predator–three-prey system and the two-predator–three-prey systems. As for the case in which foraging behaviour is faster than population dynamics (*G* ≫ 1), numerical calculations confirmed that prey species that cannot coexist in the presence of one predator species may coexist in the presence of another predator species with a different ability to discriminate prey (figure 2).

## 4. Discussion

The predator–prey model considering adaptive foraging shows that predator discrimination of prey has an important effect on the coexistence of prey species, which is in line with previous theoretical studies [5,29,30]. Specifically, it is more difficult for prey species to coexist if they belong to the same prey group of a particular predator species than if they belong to different prey groups. In fact, in a one-predator–two-prey system, when a predator species can distinguish between prey species, switching predation promotes their coexistence, and when predator species cannot distinguish between prey species, the absence of switching predation promotes apparent competition [3,8] among prey species to reduce the probability of coexistence. The same is true for the one-predator–three-prey system, as switching predation does not occur and therefore does not enhance apparent competition among prey species of the same group; hence, the prey species belonging to that group are less likely to coexist than those belonging to different groups. The conventional predator–prey models predicting that the frequent switching of predation promotes the coexistence of prey species [28] can be regarded as a special case of our model, where all the prey species belonged to different prey groups. A predator–prey model [22] which assumes another functional response (Holling's type II) and fitness function [25], yielded a qualitatively identical prediction. The prediction that prey species perceived by predators as the same prey item have difficulty coexisting appears to be independent of foraging behaviour.

The lack of species discrimination, which reduces the probability of prey coexistence, can be compensated for by another predator that is able to discriminate between members of the same prey group. A predator that cannot discriminate between some prey combinations (an imperfectly switching predator) does not promote the coexistence of those prey species. However, as shown by the mathematical model (see electronic supplementary material, appendix S5), a group of such imperfectly switching predators can allow all prey species to coexist when they are imperfect in different ways (e.g. Predator A discriminates between Prey X and Prey Y/Z, while Predator B discriminates between Prey X/Y and Prey Z). This indicates that even if all predator species fail to distinguish perfectly between different prey species, the variability in terms of how they distinguish species may promote their coexistence. In other words, a predator that can distinguish between prey species in a manner that is different from other predator species contributes to prey coexistence.

The above-mentioned prediction is relevant to the theory of the effects of Batesian mimicry [20] on multi-species coexistence. Batesian mimicry is a phenomenon in which a palatable prey mimics an unpalatable one as a model species, thus avoiding predation pressure. This theory predicts that (i) when the predator species cannot discriminate between the model and mimic, and (ii) the unpalatability is accompanied by costs, the mimic and model cannot coexist owing to the apparent competition mediated by the shared predator, or because of resource competition. Indeed, if predator species cannot discriminate between the mimic and model species, such as in perfect mimicry, and there is a difference in the growth rate between the mimic and the model, it is known that coexistence between the two is difficult [21,22]. The present study suggests that predator diversity may mitigate such difficulties in coexistence, because, although one predator species is not able to discriminate between the mimic species and the model, and therefore cannot maintain the mimicry system, the diversity in discrimination allows the persistence of the whole system.

The coexistence of prey species resulting from the different predator discrimination of prey capabilities can be explained by indirect effects among prey, which are mediated by the same predator species. These effects are strongly influenced by the group the prey belongs to. Switching prey, which affects the functional response, mediates positive effects among the prey species that belong to different groups (associated with a particular predator) to promote coexistence [11]. By contrast, apparent competition [3], which is a numerical response, causes negative effects among prey species that belong to the same group and induces species exclusion. The net effect among prey species mediated by all predator species is determined by the structure of the food web network and the type of predator discrimination of prey. For example, some prey species may not coexist owing to the negative indirect effect of predator indiscrimination; however, if there is another predator that can distinguish them from each other, the positive indirect effect will permit prey coexistence.

The present study can be extended by taking into account the complexity of real ecosystems. A possible extension of the model is to consider the case in which a prey species belongs to multiple prey groups. In this study, it was assumed that a prey species belonged to one of the prey groups of a particular predator, but never to more than one group. However, in real-world ecosystems, predator species may perceive a prey species as being part of multiple prey groups. For example, in the case of a bird preying upon the larvae and adults of a butterfly species, the group that an individual prey item belongs to may vary with its developmental stage [31]. If the intraspecific variation in prey species is large, predator species may recognize only part of the population of one prey species as another prey species (this is called imperfect mimicry) [22,25,32,33]. This poses an interesting future question regarding which interspecific interactions and community dynamics are induced by predator discrimination of prey by developmental stage, or how the diversity of imperfect mimicry impacts the community structure of prey species. Another potential extension of the model is to consider the evolution of predator discrimination of prey in predator–prey relationships. Through this extended model, it may be possible to consider the evolution of mimicry in the presence of multiple predators, as to how predator species' perception of prey species affects the mimicry process. For example, by considering the situation in which a prey species evolves to be recognized as belonging to an unpalatable prey group—thus minimizing its chances of being eaten—it may be possible to consider under what conditions prey species evolve to resemble each other (mimicry) when there are multiple predators.

It is well known that predators can act as an essential component of the prey species niche [34]. Our theoretical prediction that the coexistence of prey species is greatly affected by the predator's capability to distinguish between them suggests that the predator discrimination of prey is also an important niche component for their prey. Indeed, the present study indicates that the number of predator species does not change the coexistence potential of prey species, whereas an increase in predator discrimination of prey does.

## Data Availability

We calculated the simulation using Python (v. 3.8.9). For more detail, see the electronic supplemental material, appendix S6. The simulation code supporting this article is available from the Dryad Digital Repository: https://doi.org/10.5061/dryad.jsxksn0dg [35]. The simulation details are provided in the electronic supplementary material [36].
